# Formation of a Proton-Conducting Hydrogen-Bond Network during the L/M Transition of *Ns*XeR Uncovered by Light-Induced FTIR Spectroscopy

**DOI:** 10.1021/acs.jpclett.6c00234

**Published:** 2026-04-02

**Authors:** Yuma Ito, Kirill Kovalev, Tatsuro Nishikino, Hideki Kandori, Yuji Furutani

**Affiliations:** † Department of Life Science and Applied Chemistry, 12982 Nagoya Institute of Technology, Showa-ku, Nagoya 466-8555, Japan; ‡ European Molecular Biology Laboratory, Hamburg unit c/o DESY, Hamburg 22607, Germany; § OptoBioTechnology Research Center, 12982 Nagoya Institute of Technology, Showa-ku, Nagoya 466-8555, Japan

## Abstract

Light-driven inward proton pumping by xenorhodopsins (XeRs) challenges the view that proton-pumping microbial rhodopsins mainly generate outward proton gradients. A xenorhodopsin found from *Nanosalina* (*Ns*XeR) exhibits particularly high activity, yet the determinants of efficient inward pumping remain unclear. We used temperature-dependent light-induced FTIR difference spectroscopy to probe L and M formation beyond the K intermediate at 77 K. In L, the Schiff-base N–D stretching modes shift markedly to lower frequency, indicating a strongly hydrogen-bonded Schiff base, while an intense water O–D band emerges, consistent with assembly of a multiwater network. The carboxylic C=O stretch of Asp220 shows intermediate-specific changes that distinguish L from M and implicate Asp220 in handling protons on the cytoplasmic side. Together, the spectra support a model in which a water-mediated hydrogen-bond network organized during L-to-M formation promotes rapid proton transfer in the cytoplasmic side, consistent with a Grotthuss-type mechanism.

## Graphical abstract

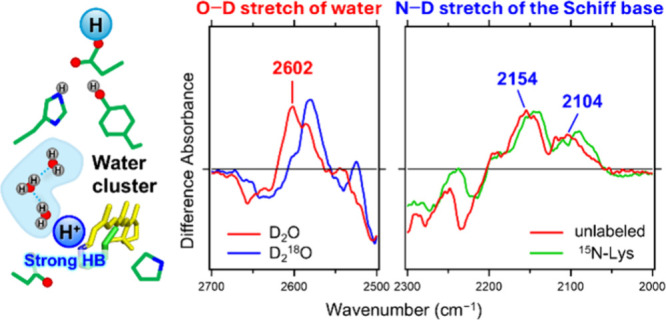

Microbial rhodopsins bind an all-*trans* retinal chromophore and express diverse light-driven functions—including ion pumps, light-gated ion channels, photosensors, and light-regulated enzymes—through photocycles initiated by retinal photoisomerization.^1^ They are membrane proteins sharing a common architecture of seven transmembrane α-helices. The retinal is covalently attached to a conserved lysine residue on helix 7 via a Schiff base, which is protonated in the dark state. The positive charge of the protonated retinal Schiff base (PRSB) is electrostatically stabilized by one or more negatively charged residues (counterions).

Light-driven proton pumps, exemplified by bacteriorhodopsin (BR) and proteorhodopsin (PR), generate a transmembrane proton-motive force by transporting protons outward, which can subsequently drive ATP synthesis.^2–4^ In these outward proton pumps, conserved acidic residues bearing carboxylate side chains in helices 3 and 7 contribute to stabilizing the PRSB (Asp85 and Asp212 in BR, Figure 1c). Upon retinal isomerization, deprotonation of the PRSB in the M intermediate—i.e., proton transfer from the PRSB to a conserved aspartate located in helix 3—is a key chemical step underlying outward proton transport.

**Figure 1:**
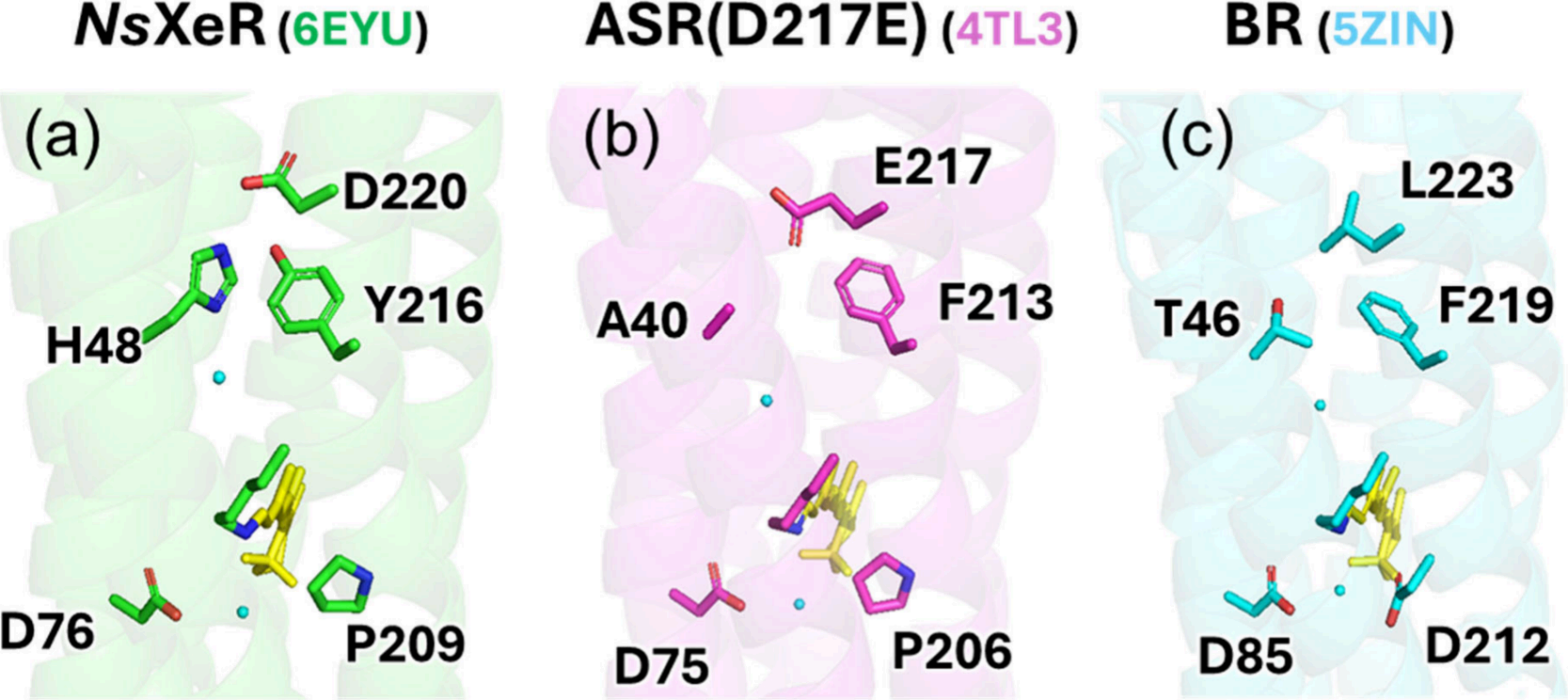
X-ray crystal structures of *Ns*XeR (a), ASR­(D217E) (b), and BR (c). The PDB ID for each structure is given in parentheses. The retinal chromophore is shown in yellow. Small spheres represent oxygen atoms of water molecules.

In contrast, microbial rhodopsins functioning as inward proton pumps were long thought not to exist in nature because inward pumping would dissipate the proton-motive force. In this context, the discovery that the D217E mutant of Anabaena sensory rhodopsin (ASR) exhibits inward proton-pumping activity was striking.^5^ Contrary to the prevailing assumption, naturally occurring inward proton pumps have since been identified, including xenorhodopsins (XeRs).^6,7^ More recently, schizorhodopsins (SzRs), another class of light-driven inward proton pumps, were found in Asgard archaea.^8^ The growing number of inward proton pumps discovered across diverse microorganisms has renewed interest in their physiological roles, which remain largely unclear.

Several structural features have been proposed as hallmarks of inward proton pumps. In many xenorhodopsins, the acidic residue (Asp) that typically serves as a counterion in helix 7 is replaced by Pro (Pro209 in *Ns*XeR, Figure 1a). In schizorhodopsins, by contrast, the canonical counterion position in helix 3 is occupied by a neutral aromatic residue such as Phe or Tyr. Thus, a common theme among inward proton pumps is stabilization of the protonated RSB by a single negative charge rather than by two acidic counterions.

SzRs also exhibit a distinct sequence of thermal reisomerization events compared with outward proton pumps. In BR and PR, photoisomerization converts the retinal from all-*trans* to 13-*cis*, followed by proton transfer from the PRSB to the counterion (e.g., Asp85 in BR or Asp97 in PR), yielding the M intermediate (Figure 1c). Subsequent reprotonation of the RSB from a cytoplasmic donor residue (e.g., Asp96 in BR or Glu108 in PR) leads to formation of later intermediates, and the retinal then thermally reisomerizes from 13-*cis* back to all-*trans* to complete the photocycle. In other words, in outward proton pumps, thermal reisomerization to all-*trans* occurs after the key proton-transfer steps. In SzRs, M is likewise formed after transfer of the Schiff-base proton toward the cytoplasmic side; however, the retinal thermally reisomerizes from 13-*cis* to all-*trans* before proton uptake from the extracellular side.^9,10^ This order of events has been proposed to be rational for inward proton transport.

For XeRs, a branched model has been reported in which the 13-*cis*,15-*anti* configuration generated by photoisomerization proceeds either directly to all-*trans* by thermal isomerization or via a 13-*cis*,15-*syn* intermediate formed by isomerization around C_15_, followed by a concerted thermal isomerization around C_13_ and C_15_ to regenerate all-*trans*.^7,11^ In either route, the retinal Schiff base is thought to undergo thermal isomerization while deprotonated, and only subsequently to be reprotonated from the extracellular side. This sequence would allow the nitrogen lone pair of the Schiff base to be oriented toward the extracellular side upon reprotonation, providing a mechanistic rationale for inward proton pumping.^12^

Transfer of the Schiff-base proton toward the cytoplasmic side is essential for inward proton-pumping activity. Accordingly, the identity of the primary proton acceptor and the pathway of proton transfer have been extensively investigated by infrared spectroscopy. In the first-discovered *Po*XeR and in the engineered ASR­(D217E) mutant, Asp216 and Glu217 in helix 7 have been reported to act as the primary proton acceptor, respectively (Figure 1b).^7,13^ In *Ns*XeR, however, two independent studies have concluded that the corresponding Asp220 (Figure 1a) is not the initial proton acceptor.^14,15^ Time-resolved infrared measurements on *Ns*XeR suggested that the Schiff-base proton is not transferred directly to Asp220, implying the presence of an intermediate acceptor (a “proton donor/relay” in subsequent steps) preceding Asp220. His48 has been proposed as a candidate primary acceptor, but direct experimental evidence is still lacking because none of the 19 possible substitutions at this position yielded functional protein.^14,16^

The involvement of internal water molecules is also critical for proton transfer from the protonated RSB, as demonstrated for both outward and inward proton pumps. For outward proton pumps such as BR, Kandori et al. have proposed that strongly hydrogen-bonded water molecules located near the PRSB play indispensable roles in directing and enabling proton transfer.^17–21^ More recently, resonance Raman spectroscopy of the inward proton pump SzR4 suggested that formation of a strongly hydrogen-bonded interaction between the PRSB and a water molecule in the L intermediate is important for determining the directionality of proton transport.^22^ In a newly identified bacterial XeR, *Bc*XeR from *Bacillus coahuilensis* as well, time-resolved and low-temperature-trapped X-ray crystallographic analyses of the L and M intermediates revealed that, in the L intermediate, three water molecules form an approximately linear hydrogen-bond network between the PRSB and Ser211 on the cytoplasmic side (Figure S1).^23^ Similar water-network formation has also been reported in BR, and these observations collectively underscore the potential importance of Grotthuss-type proton translocation through hydrogen-bonded water chains, in which protons are transferred by hopping along the hydrogen-bond network without requiring diffusion of the water molecules themselves.^24–27^

In the present study, we employed low-temperature infrared spectroscopy to analyze spectral changes accompanying the formation of the L and M intermediates of *Ns*XeR, focusing on (i) the carboxylic C=O stretching region reporting on protonated carboxyl groups, (ii) the O–D stretching region of internal water molecules, and (iii) the N–D stretching region of the PRSB. This study provides the first comprehensive vibrational characterization of the key elements governing proton transfer on the cytoplasmic side of an inward proton pump rhodopsin. High-precision measurements in the carboxylic C=O stretching region establish definitive assignments of protonated carboxyl groups, resolving inconsistencies in earlier reports.^14,15^ In addition, the O–D stretching region reveals strong signatures of internal water molecules, while isotope-edited N–D stretching bands demonstrate formation of a strongly hydrogen-bonded PRSB in the L intermediate. Together, these results identify the cooperative roles of internal waters and the Schiff base in mediating proton transfer toward the cytoplasmic side, providing the first direct spectroscopic evidence for the proton-conducting hydrogen-bond network underlying efficient inward proton pumping in *Ns*XeR.

## Comparison of UV–Vis and FTIR Spectra under Identical Temperature Conditions to Identify Temperatures Suitable for Trapping the L and M Intermediates

The light-induced FTIR difference spectra corresponding to the L and M intermediates of *Ns*XeR were obtained by illuminating liposome-reconstituted samples under cryogenic conditions between 77 and 260 K. As shown in Figure S2, UV–vis absorption spectra indicate that the K intermediate is predominantly stabilized at 77 K, the L intermediate at 170 K, and the M intermediate at 230 K. The FTIR difference spectra (Figure S3) exhibited clear temperature-dependent changes: the spectral pattern altered upon increasing the temperature from 77 K to 170 and 200 K, and additional changes emerged at 220, 230, and 260 K. The presence of three major stages of spectral evolution is consistent with predominant stabilization of K at 77 K, L at 170 K, and M at 230 K (The representative infrared spectra are shown in Figure 2a as well). C–C stretching vibrations and HOOP (hydrogen-out-of-plane) vibrations are also useful for evaluating the retinal structure in the L and M intermediates. The light-induced FTIR difference spectra of the WT measured at 77, 170, and 230 K are shown in Figure S4a for the C–C stretching region and in Figure S5a for the HOOP region. The K intermediate (77 K) exhibits a C–C stretching band at 1191 cm^–1^, characteristic of the 13-*cis* retinal configuration. In the L intermediate (170 K), the corresponding band appears at 1188 cm^–1^ and shows an H/D isotope effect. In the M intermediate (230 K), the C–C stretching band nearly disappears due to deprotonation of the retinal Schiff base.

**Figure 2:**
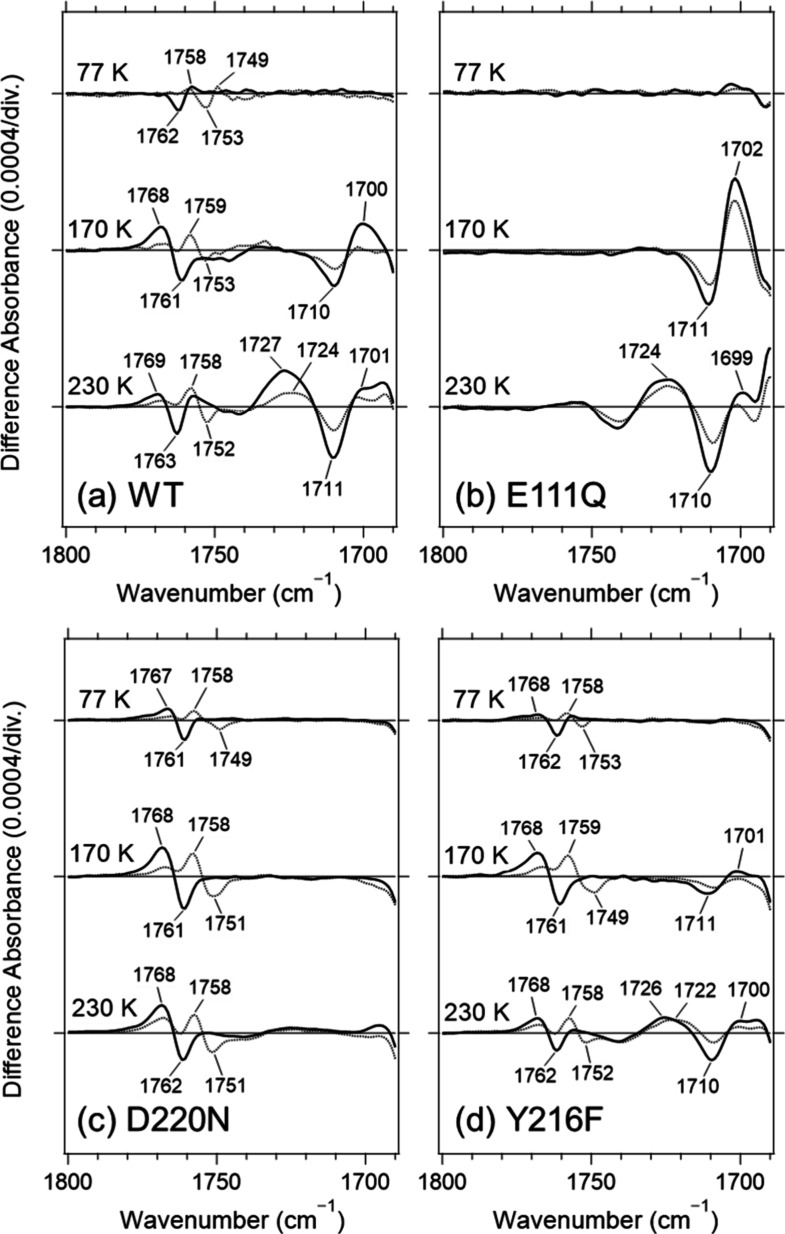
Light-induced FTIR difference spectra in the 1800–1690 cm^–1^ region recorded at 77, 170, and 230 K for WT (a), E111Q (b), D220N (c), and Y216F (d). Solid and dotted lines denote spectra measured under H_2_O and D_2_O hydration, respectively. One division on the *y*-axis corresponds to 0.0004 absorbance units in each panel.

The HOOP region provides information on retinal distortion. The band at 1009 cm^–1^, which appears in the K intermediate (77 K) and exhibits an H/D isotope effect, disappears in the L and M intermediates, whereas the band at 953 cm^–1^ becomes slightly more intense in the L intermediate. These bands are useful spectral markers for distinguishing the K, L, and M intermediates.

## Comparison of the Carboxylic C=O Stretching Vibrations in the K, L, and M Intermediates

At 77 K, a pair of oppositely signed bands appeared at (−) 1762 and (+) 1758 cm^–1^ (Figure 2a). Upon D_2_O hydration, these bands shifted to lower frequencies to (−) 1753 and (+) 1749 cm^–1^, suggesting that they originate from the C=O stretching vibration of a protonated carboxylic group (Asp or Glu). At 170 K, the band pair shifted to higher frequency, yielding a pattern centered at (+) 1768 and (−) 1761 cm^–1^. At 230 K, the positive component exhibited a slight additional upshift to approximately (+) 1769 cm^–1^, while its intensity decreased and a distinct positive band emerged near 1758 cm^–1^. In earlier time-resolved FTIR studies, the band pair observed in the wild type at (+) 1771/(−) 1763 cm^–1^ underwent a large downshift to (+) 1703/(−) 1695 cm^–1^ in the E111Q mutant, leading to assignment of this feature to the carboxyl vibration of Glu111.

In addition, a band pair at (−) 1710 and (+) 1700 cm^–1^ became prominent at 170 K. Because its intensity was strongly attenuated under D_2_O hydration, this feature is also attributable to the C=O stretching vibration of an Asp or Glu carboxylic group. At 230 K, the intensity of the ∼1700 cm^–1^ band decreased, whereas a new positive band near 1727 cm^–1^ grew in intensity. The 1727 cm^–1^ band reached its maximum at 230 K, suggesting that the spectrum at 260 K contains an increased contribution from a subsequent intermediate, plausibly an M_2_-like state (Figure S3b).

Previous time-resolved FTIR studies have reported differing interpretations for the Asp220-associated signals. Schubert et al. assigned the negative band at 1711 cm^–1^ to Asp220 and concluded that Asp220 is deprotonated in the L and M_1_ intermediates because the corresponding feature is absent in those states.^14^ In contrast, Asido et al. likewise assigned a negative band at 1710 cm^–1^ to Asp220, but reported the appearance of a positive band at 1745 cm^–1^ in the temporal window corresponding to the K and L intermediates and a positive band at 1726 cm^–1^ in the M intermediate.^15^

## Assignment of the Glu111 and Asp220 Carboxylic Bands in Low-Temperature FTIR Difference Spectra

The 1800–1690 cm^–1^ region, which reports on C=O stretching vibrations of carboxylic acids, has previously been discussed for *Ns*XeR mainly on the basis of time-resolved FTIR measurements.^14,15^ Here, we newly performed band assignments in low-temperature FTIR difference spectra. The spectra trapped at 77, 170, and 230 K were correlated with predominant accumulation of the K, L, and M intermediates, respectively (Figure 2a), which were supported by the C–C and HOOP vibrational regions (Figure S4 and S5) and the UV–vis spectra recorded at the same temperatures (Figure S2) as already discussed.

First, we studied the E111Q mutant. In this mutant, the characteristic band pairs observed in the wild type—(−) 1762/(+) 1758 cm^–1^ in K (77 K), (+) 1768/(−) 1761 cm^–1^ in L (170 K), and (+) 1769/(−) 1763 cm^–1^ in M (230 K)—were cleanly abolished (Figure 2b). The intermediates stabilized at each temperature were confirmed by low-temperature UV–visible spectroscopy (Figure S6) as well as by characteristic C–C stretching and HOOP vibrational bands (Figure S4 and S5). We therefore conclude that these features originate from the C=O stretching vibration of the Glu111 carboxylic group. In contrast, the positive band near 1758 cm^–1^ at 230 K appeared to remain, at least in part, in the E111Q mutant, suggesting that this band may arise from a different carboxylic group.

Next, we examined the D220N mutant. In this mutant, the band pair observed in the wild-type L intermediate at (−) 1710/(+) 1700 cm^–1^, as well as the band pair observed in the M intermediate at (+) 1727/(−) 1711 cm^–1^, disappeared nearly completely (Figure 2c). Accordingly, we assign these features to the C=O stretching vibration of the Asp220 carboxylic group. A small residual positive contribution around 1690 cm^–1^ at 230 K may reflect an additional carboxylic group. In earlier time-resolved FTIR studies, a band near 1693 cm^–1^ was tentatively suggested to originate from Asp76,^15^ a candidate proton donor to the RSB; however, a reliable assignment remains an open question for future work. In the D220N mutant, the L intermediate absorbing at 480 nm remains even at 230 K (Figure S6b), and the HOOP band at 953 cm^–1^ also exhibits relatively high intensity (Figure S5c). These observations indicate that formation of the M intermediate is inhibited at 230 K in this mutant. However, this effect does not significantly affect the assignment of the carboxylic C=O stretching bands.

Taken together, the present results provide, to our knowledge, the first assignments of the Glu111 and Asp220 carboxylic C=O stretching signals in low-temperature FTIR difference spectra of *Ns*XeR. While the assignment of Glu111 is consistent with conclusions from previous time-resolved FTIR studies,^14^ the behavior of Asp220 differs among reports.^14,15^ In our low-temperature spectra, the Asp220 C=O band shifts to lower frequency (∼1700 cm^–1^) in the L intermediate, consistent with strengthened hydrogen bonding, whereas it shifts to higher frequency (∼1727 cm^–1^) in the M intermediate, consistent with weakened hydrogen bonding. Under our conditions, we did not observe a spectral signature corresponding to a deprotonated Asp220 as reported by Schubert et al.,^14^ nor did we detect the strongly upshifted feature near 1745 cm^–1^ reported by Asido et al.,^15^ which would indicate a very weakly hydrogen-bonded environment. These discrepancies may reflect differences between room-temperature time-resolved FTIR measurements and low-temperature trapping approaches that stabilize intermediates under cryogenic conditions. In addition, Asido et al. analyzed detergent-solubilized samples in DDM, whereas our measurements were performed on liposome-reconstituted *Ns*XeR, which may further contribute to the observed differences.

We further examined the effect of a Tyr-to-Phe substitution at position 216 (Y216F), located in the vicinity of Asp220 (Figure 1a). In the Y216F mutant, formation of the K, L, and M intermediates was essentially identical to that observed in the wild type (Figure S4d, S5d, and S6c). The Y216F mutation had little influence on the Glu111-associated C=O bands, whereas it markedly reduced the intensity of the Asp220-associated features (Figure 2d). Because the peak positions were largely unchanged, Tyr216 is unlikely to participate directly in the hydrogen bonding that determines the Asp220 C=O frequency; instead, the mutation appears to modulate the Asp220 signal indirectly, for example by altering local electrostatics, packing, or the population of the trapped intermediate(s).

## Assignment of the N–D Stretching Vibration of the Retinal Schiff Base and the O–D Stretching Vibrations of Internal Waters

Hydrogen bonding of the protonated retinal Schiff base (PRSB) and the hydrogen-bond network formed by internal water molecules provide key information for understanding proton-transfer reactions.^21^ In particular, the 2800–2000 cm^–1^ region, which contains the N–D stretching vibration of the Schiff base and the O–D stretching vibrations of bound waters, serves as a sensitive probe of hydrogen-bond strength (Figure 3). In our previous study, we reported that, in the K intermediate trapped at 77 K, the Schiff-base N–D stretching band undergoes a large upshift from 2237 cm^–1^ in the dark state to 2475 cm^–1^ (Figure 3a), indicating that formation of K markedly weakens the hydrogen bonding of the PRSB in *Ns*XeR.^28^ We also observed concomitant changes in the O–D stretching region, with bands at (−) 2654, (+) 2637, (−) 2628, (+) 2617, and (+) 2561 cm^–1^, which were attributed to internal water molecules (Figure 3a).^28^

**Figure 3:**
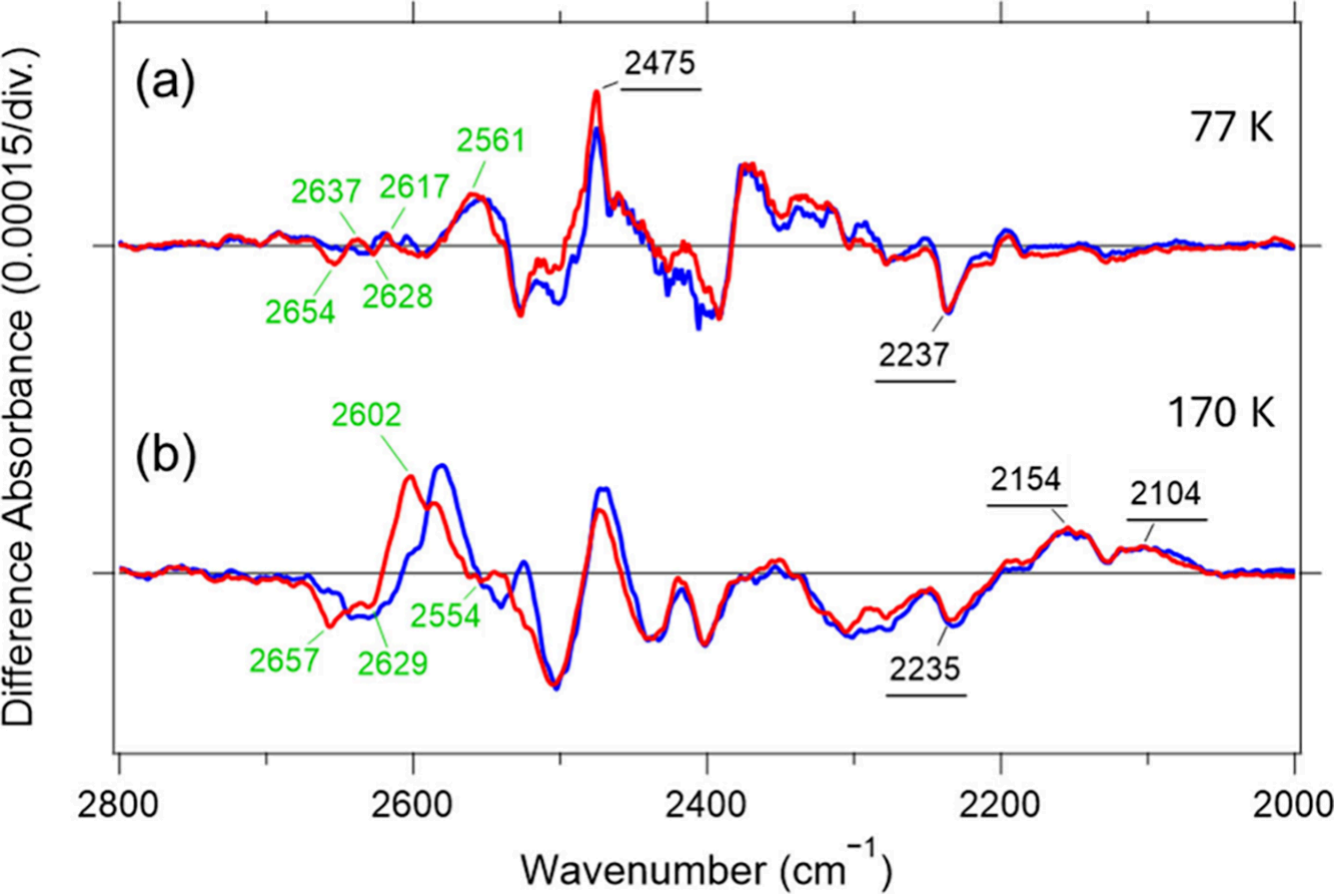
Light-induced FTIR difference spectra in the 2800–2000 cm^–1^ region recorded at 77 K (a) and 170 K (b). Red and blue solid lines denote spectra measured under D_2_O and D_2_^18^O hydration, respectively. One division on the *y*-axis corresponds to 0.00015 absorbance units. The K-minus-*Ns*XeR spectra are reproduced with permission from the ref 28. Copyright 2025 Elsevier.

To extend this analysis to the L intermediate, we performed analogous measurements under conditions where L is predominantly stabilized (170 K). The resulting FTIR difference spectrum is shown in Figure 3b. By comparing spectra of D_2_O-hydrated samples (red trace) with those recorded under D_2_^18^O hydration (blue trace), we assigned the bands at (−) 2657, (−) 2629, (+) 2602, and (−) 2554 cm^–1^ to O–D stretching vibrations of internal water molecules. Notably, the positive band at 2602 cm^–1^ was substantially more intense than the corresponding O–D features observed for the K intermediate. This observation suggests contributions from multiple water molecules and/or from a water molecule placed in a highly polar environment that enhances the effective transition dipole moment. Interestingly, a similarly intense water O–D stretching band has also been reported at 2596 cm^–1^ in the L intermediate of BR, a light-driven outward proton pump (Figure S7).^18^ The possible relevance of this feature to proton transfer is discussed later.

The negative band at 2235 cm^–1^ is closely consistent with the negative band at 2237 cm^–1^ observed at 77 K, supporting its assignment to the N–D stretching vibration of the Schiff base in the dark state. In the L intermediate trapped at 170 K, new positive bands appeared at (+) 2154 and (+) 2104 cm^–1^.

To assign these N–D stretching bands, we conducted the same measurements using ^15^N-labeled samples: a uniformly ^15^N-labeled protein (^15^N-uniform) and a sample specifically labeled at the ε-nitrogen of lysine residue (^15^N-ε-Lys) (Figure 4). In the ^15^N-uniform sample, the bands at (−) 2235, (+) 2154, and (+) 2104 cm^–1^ shifted to (−) 2221, (+) 2148, and (+) 2091 cm^–1^, respectively. In the ^15^N-ε-Lys sample, they shifted similarly to (−) 2221, (+) 2145, and (+) 2091 cm^–1^. These consistent downshifts demonstrate that all three bands originate from N–D stretching vibrations involving the Schiff-base nitrogen of the retinal-binding lysine. Accordingly, we assign the PRSB N–D stretching bands in the L intermediate to 2154 and 2104 cm^–1^, indicating that the Schiff base forms stronger hydrogen bonding in L than in the dark state.

**Figure 4:**
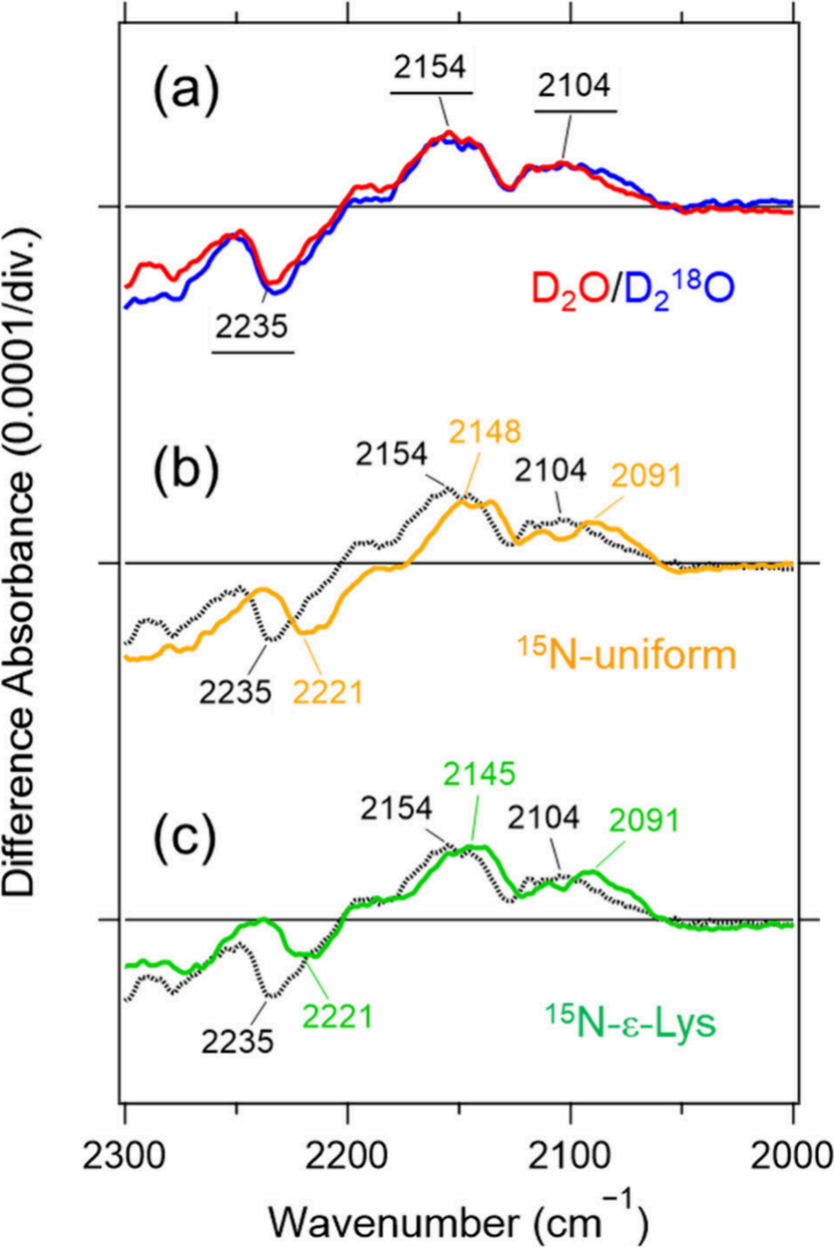
Light-induced FTIR difference spectra in the 2300–2000 cm^–1^ region recorded at 170 K for comparison of spectra with D_2_^18^O (a), ^15^N-uniform (b), and ^15^N-ε-Lys (c) with the D_2_O spectrum. Red and blue solid lines in (a) denote spectra measured under D_2_O and D_2_^18^O hydration, respectively. Orange and green colored lines in (b) and (c) denote spectra measured ^15^N-unifrom and ^15^N-ε-Lys samples under D_2_O hydration, respectively. The black dotted lines are reproduced from the red spectrum in (a). One division on the *y*-axis corresponds to 0.0001 absorbance units.

We further examined whether mutations near Asp220 influence the O–D stretching bands of internal waters or the PRSB N–D stretching bands (Figure 5). Both D220N and Y216F mutants exhibited spectral changes essentially identical to those of the wild type, indicating that these O–D and PRSB N–D features are not strongly perturbed by these substitutions in either the dark state or upon L formation. This is consistent with X-ray crystal structures showing that Asp220 and Tyr216 are located at a distance from the PRSB (Figure 1a and Figure S1a),^16^ making direct interaction through a shared hydrogen-bond network or through the protein backbone unlikely. These results also suggest that the water molecule(s) responsible for the intense O–D stretching band observed in the L intermediate do not reside near Asp220 or Tyr216 and are unlikely to be part of a hydrogen-bond network in that region.

**Figure 5:**
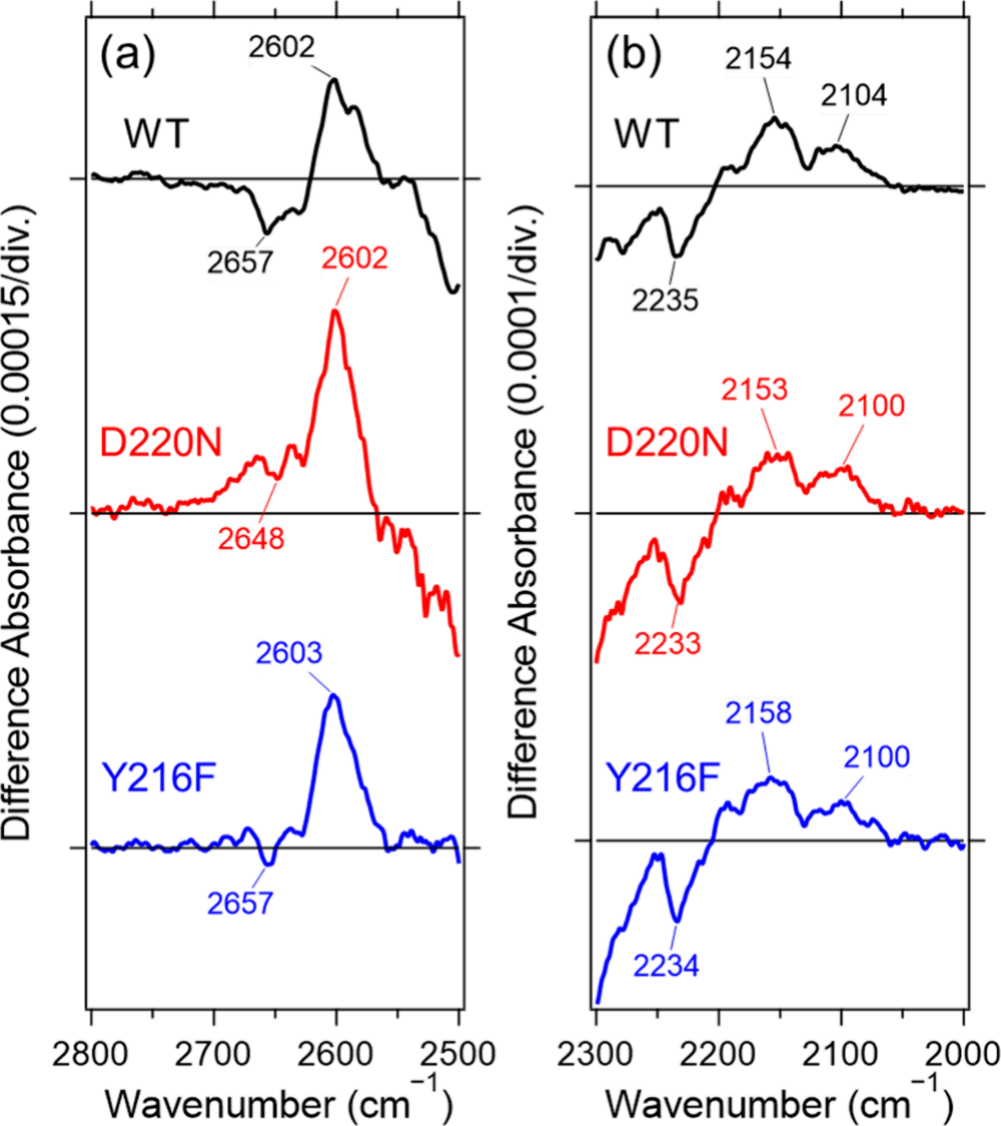
Light-induced FTIR difference spectra in the 2800–2500 cm^–1^ (a) and 2300–2000 cm^–1^ (b) regions recorded at 170 K for WT, D220N, and Y216F. The spectra of WT, D220N, and Y216F were measured under D_2_O hydration. One division on the *y*-axis corresponds to 0.00015 (a) and 0.0001 (b) absorbance units.

To assess whether analogous water-related O–D features are observed in other XeRs, we performed the same measurements on *Bc*XeR, for which the structure of the L intermediate has been determined by X-ray crystallography.^23^ The FTIR difference spectra of *Bc*XeR revealed O–D stretching bands at (−) 2637, (+) 2581, and (−) 2549 cm^–1^, attributable to internal water molecules (Figure S8). Notably, the positive band at 2581 cm^–1^ was also unusually intense, resembling the strong O–D feature observed in *Ns*XeR. The low-temperature-trapped crystal structure of the *Bc*XeR L intermediate shows that three water molecules form an approximately linear hydrogen-bond network between the PRSB and Ser211 (Figure S1c).^23^ The intense O–D band observed here may therefore be related to these reorganized internal waters, potentially reflecting formation or strengthening of a water-mediated hydrogen-bond network in the L state.

In contrast, the 2596 cm^–1^ water O–D stretching band observed in BR (Figure S7) has been correlated with an intense 3490-cm^–1^ band in the O–H stretching region, and the band intensity is substantially diminished in the T46 V (H48 in *Ns*XeR) and V49 M (I51 in *Ns*XeR) mutants.^29^ In addition, the O–H stretching band was slightly downshifted in the D96N mutant (A87 in *Ns*XeR).^29^ The water molecules in the cytoplasmic region in BR forms a hydrogen-bonding network different from that of *Ns*XeR, although the band shape and the wavenumber are similar to those of *Ns*XeR.

## Proposed Proton-Transfer Mechanism from the PRSB via Water Molecules and Asp220 in NsXeR

Based on the low-temperature FTIR signatures obtained in this study—namely, the C=O stretching vibrations of the Asp220 carboxylic group, the N–D stretching vibration of the PRSB, and the O–D stretching vibrations of internal water molecules—we propose the following scheme for proton-transfer events on the cytoplasmic side during the formation of the M intermediate from the L intermediate (Figure 6).

**Figure 6:**
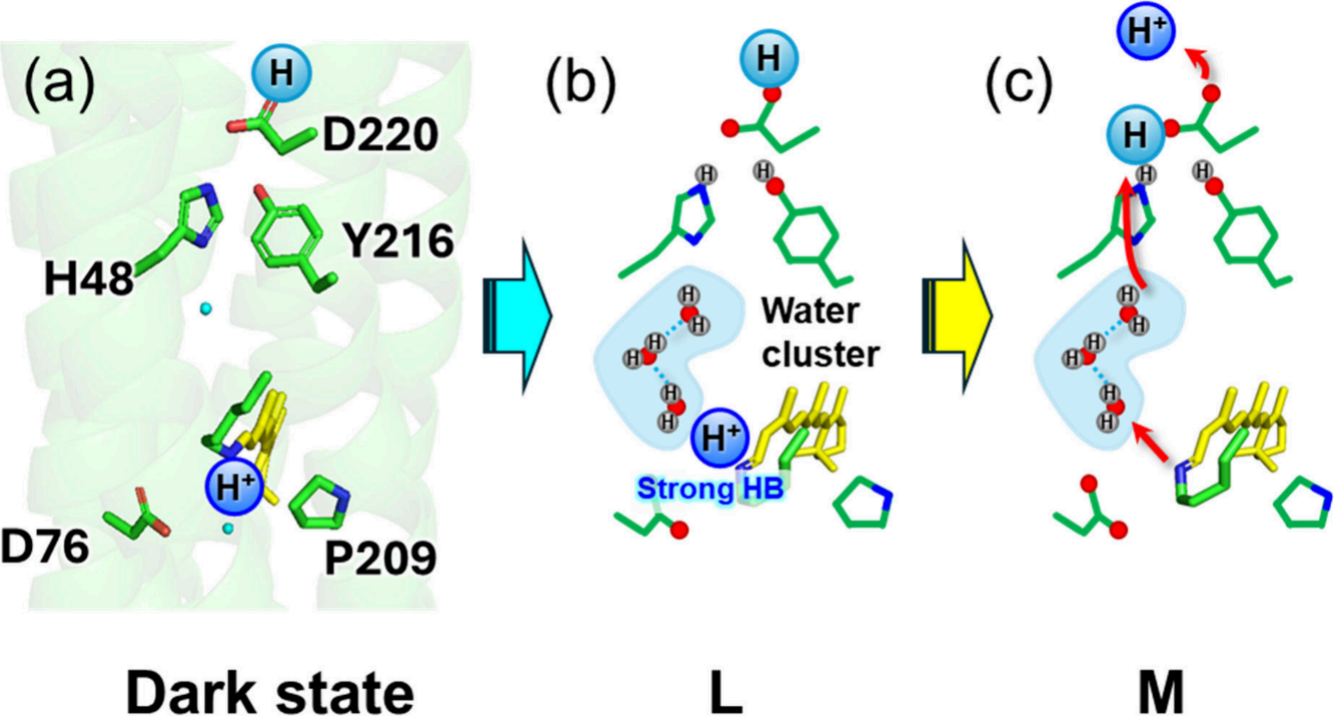
Schematic models for proton-transfer reactions in the cytoplasmic region of *Ns*XeR. (a) Dark-state structure rendered in PyMOL based on the X-ray crystal structure (PDB ID: 6EYU). (b) Schematic model of the L intermediate inferred from this study: the Schiff-base proton is oriented toward the cytoplasmic side and forms a strong hydrogen bond, accompanied by formation of a water cluster along the proton-conduction pathway. (c) Schematic model of the M intermediate, illustrating proton transfer toward the cytoplasmic bulk via the water cluster. HB is abbreviation of hydrogen bond.

First, retinal photoisomerization from the all-*trans* configuration to the untwisted 13-*cis* configuration in the K intermediate suggests that the PRSB proton becomes oriented toward the cytoplasmic side. At the same time, the PRSB hydrogen bond is markedly weakened, as evidenced by the large upshift of the Schiff-base N–D stretching vibration previously observed for K.^28^

In contrast, upon formation of the L intermediate, our present results demonstrate that the PRSB forms a stronger hydrogen bond than in the dark state. Resonance Raman studies of the inward proton pump SzR4 have similarly reported that the PRSB forms a strong hydrogen bond in the L intermediate and that a water molecule is likely the interaction partner.^22^ For *Ns*XeR, while our data clearly indicate strengthened hydrogen bonding at the PRSB in L, we did not obtain direct spectroscopic evidence that uniquely identifies water as the hydrogen-bond partner. Nevertheless, our observations can be naturally related to the L-intermediate crystal structure of *Bc*XeR,^23^ where the PRSB is positioned to hydrogen-bond with an internal water molecule and, moreover, additional two water molecules form an approximately linear hydrogen-bond network extending toward Ser211 on the cytoplasmic side (Figure S1). Consistent with this structural picture, an unusually intense O–D stretching band attributable to internal waters was observed in the L intermediate of both *Ns*XeR and *Bc*XeR (Figure S8). It is therefore plausible that the water bands detected here reflect a rearranged water cluster corresponding to the three-water chain captured crystallographically in *Bc*XeR.^23^ On the other hand, the essentially unchanged N–D and O–D spectral features in the D220N and Y216F mutants suggest that the PRSB-associated hydrogen-bond network does not extend to the vicinity of Asp220 or Tyr216 but is interrupted upstream. This interpretation is consistent with previous time-resolved FTIR studies proposing that, in *Ns*XeR, the PRSB proton is not transferred directly to Asp220 upon M formation.^14,15^

We therefore suggest that, during the L-to-M transition, the PRSB proton is first accepted by His48 or by another primary proton acceptor on the cytoplasmic side, while the proton initially residing on Asp220 is released to the cytoplasmic bulk. In a subsequent step, a proton is transferred from the primary acceptor to Asp220, completing the inward proton-transfer sequence associated with M formation.

Such a multistep proton-transfer mechanism is expected to account for the pronounced difference in the kinetics of M formation in H_2_O versus D_2_O, as reflected by the large kinetic isotope effect (KIE ≈ 4) for the L-to-M transition.^14^

The cytoplasmic region of *Bc*XeR is highly similar to that of *Ns*XeR (Figure S1); however, in *Bc*XeR the residue corresponding to His48 is Ala, and therefore it cannot function as a proton acceptor of the PRSB. Interestingly, previous studies have reported that substitution of His48 in *Ns*XeR with any of the other 19 amino acids did not yield functional protein.^16^ In contrast, introducing a His residue at the corresponding position in *Bc*XeR (A38H) increases the proton-pumping activity by approximately 1.7-fold.^23^ These observations further support the importance of His48 in achieving the high inward proton-pumping activity of *Ns*XeR.

In conclusion, this study establishes a compact, band-assigned picture of L- and M-state dynamics in the inward proton pump *Ns*XeR using low-temperature trapping FTIR combined with mutagenesis and isotope labeling. Stabilization of K (77 K), L (170 K), and M (230 K) enabled intermediate-resolved comparison and, critically, opened the whole mid-infrared window for cryo-trapped *Ns*XeR intermediates. Glu111 and Asp220 carboxylic C=O stretches were assigned by the selective loss of signals in E111Q and D220N, revealing that Asp220 experiences stronger hydrogen bonding in L and weaker hydrogen bonding in M. In parallel, the L state shows a large downshift of the PRSB N–D stretching modes to 2154 and 2104 cm^–1^ (confirmed by ^15^N-uniform and ^15^N-ε-Lys labels), together with an intense water O–D stretch near 2602 cm^–1^ (confirmed by D_2_^18^O hydration), indicating that L organizes a highly hydrogen-bonded, water-rich environment. Integrating these markers with prior kinetic observations, we propose that L formation assembles a proton-conducting water network that supports efficient proton translocation, with proton acceptance occurring first at an upstream site including a water cluster and subsequent involvement of Asp220. Overall, *Ns*XeR achieves inward pumping through staged remodeling of the Schiff base, internal waters, and Asp220.

## Supplementary Material



## Notes

X-ray crystal structures of *Bc*XeR and *Ns*XeR comparing the cytoplasmic sides, light-induced UV–visible and FTIR difference spectra of *Ns*XeR WT, E111Q, D220N, and Y216F mutants recorded at 77–260 K, light-induced difference FTIR spectra of *Ns*XeR WT, BR, and *Bc*XeR in the O–D and N–D stretching region recorded at 170 K, and description of the materials and methods used in this study (PDF)

## Glossary

PRSB: protonated retinal Schiff base
XeR: xenorhodopsin
SzR: schizorhodopsin
BR: bacteriorhodopsin
FTIR: Fourier transform infrared.
