# Supplementary material for: Formation of a Proton-Conducting Hydrogen-Bond Network during the L/M Transition of NsXeR Uncovered by Light-Induced FTIR Spectroscopy
Source: J Phys Chem Lett. 2026 Apr 2;17(15):4399–407. doi: 10.1021/acs.jpclett.6c00234 (PMC13093656; doi:10.1021/acs.jpclett.6c00234)
Supplement: Supplementary file 1 [file jz6c00234_si_001.pdf]

Supporting Information

Formation of a Proton-Conducting Hydrogen-Bond Network During the L/M Transition of *Ns*XeR Uncovered by Light-Induced FTIR Spectroscopy

Yuma Ito[1], *Kirill Kovalev*[2], *Tatsuro Nishikino*[1 3], *Hideki Kandori*[1,3], *and Yuji Furutani*[1,3]*

[1]Department of Life Science and Applied Chemistry, Nagoya Institute of Technology, Showa-ku, Nagoya 466-8555, Japan

[2]European Molecular Biology Laboratory, Hamburg unit c/o DESY, Hamburg, Germany

[3]OptoBioTechnology Research Center, Nagoya Institute of Technology, Showa-ku, Nagoya 466-8555, Japan

* Address Correspondence to Phone & Fax: +81 52 735 5127, E-mail: furutani.yuji@nitech.ac.jp

List of the contents in the supporting information

Figure S1: Comparison of the X-ray crystal structures of the dark states of *Ns*XeR and *Bc*XeR and the L intermediate of *Bc*XeR.

Figure S2: Light-induced difference visible absorption spectra of *Ns*XeR WT recorded at 77–260 K.

Figure S3: Light-induced difference FTIR spectra of *Ns*XeR WT recorded at 77–260 K.

Figure S4. Light-induced FTIR difference spectra in the 1300–1100 cm^{-1} region recorded at 77, 170, and 230 K for WT (a), E111Q (b), D220N (c), and Y216F (d).

Figure S5. Light-induced FTIR difference spectra in the 1050–900 cm^{-1} region recorded at 77, 170, and 230 K for WT (a), E111Q (b), D220N (c), and Y216F (d).

Figure S6. Light-induced difference visible absorption spectra of E111Q, D220N, and Y216F mutants of *Ns*XeR recorded at 77, 170, and 230 K.

Figure S7: Comparison of light-induced difference FTIR spectra of *Ns*XeR and BR in the O–D and N–D stretching region recorded at 170 K.

Figure S8: Comparison of light-induced difference FTIR spectra of *Ns*XeR and *Bc*XeR in the O–D stretching region recorded at 170 K.

Description of materials and methods used in this study

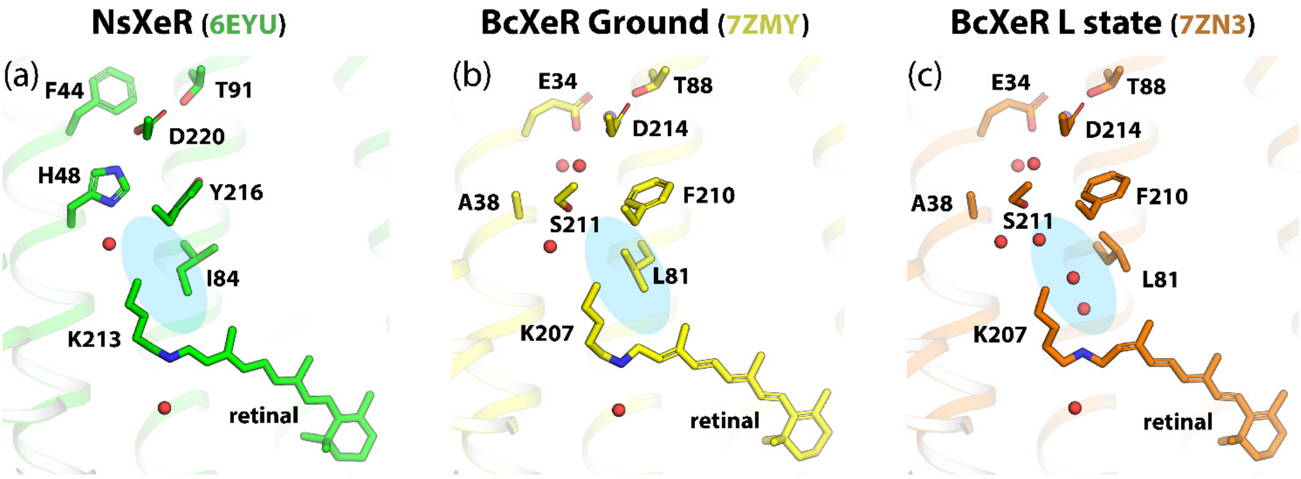

Figure S1. Comparison of the X-ray crystal structures of the dark states of *Ns*XeR (a) and *Bc*XeR (b) and the L intermediate of *Bc*XeR (c). The PDB ID for each structure is given in parentheses. Small red spheres represent oxygen atoms of water molecules. The light blue ellipses indicate cavities that accommodate water molecules.

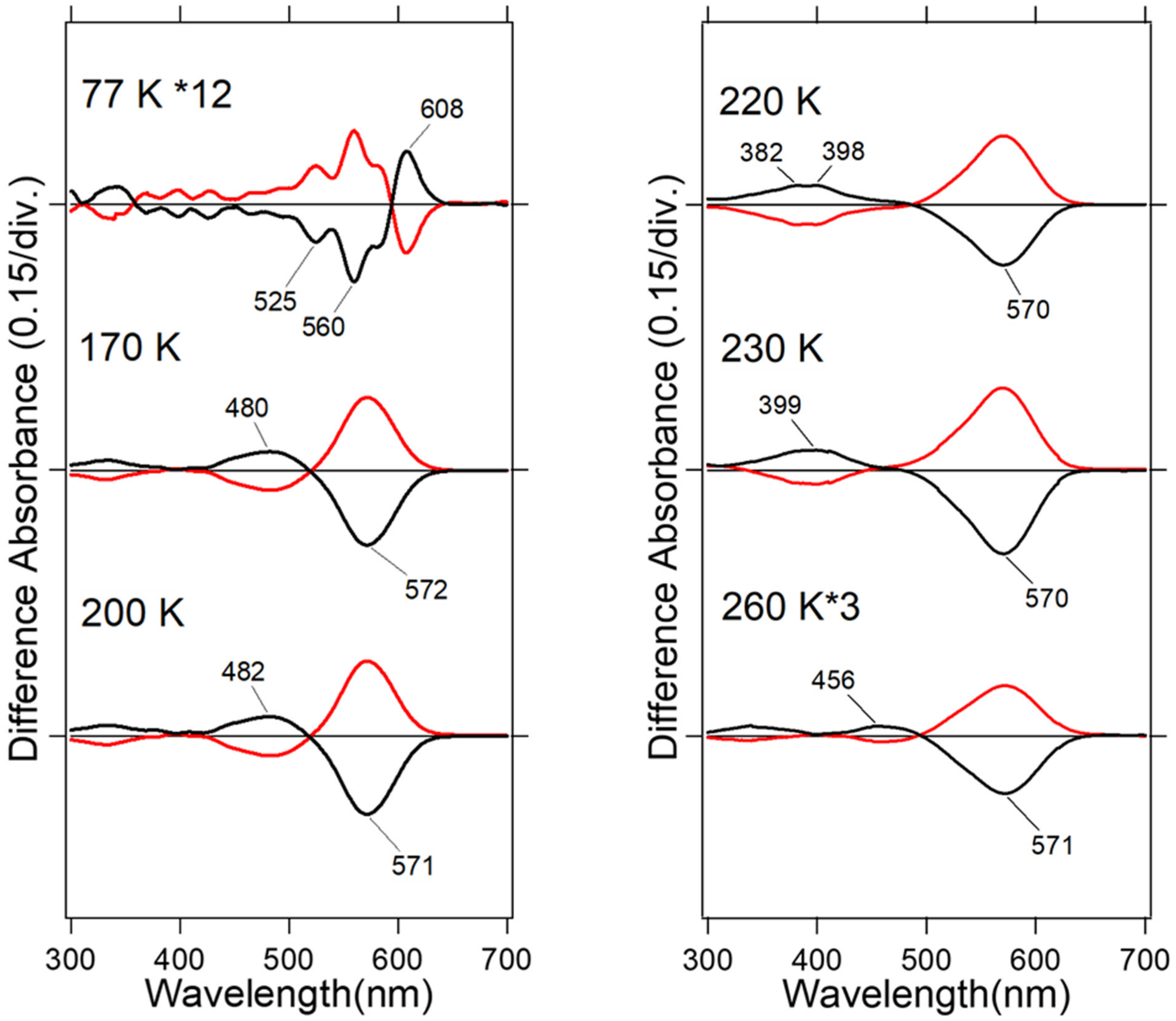

Figure S2. Light-induced difference visible absorption spectra of *Ns*XeR recorded at 77–260 K. The forward and backward photoreactions are represented by solid black and red lines, respectively. In each panel, the difference spectrum (black line) shows the negative and positive sides corresponding to the dark state and the red-shifted (K) or blue-shifted (L or M) intermediate, respectively. The difference spectrum (red line) in each panel represents the reverse reaction. The spectra recorded at 77 and 260 K were normalized by factors of 12 and 3, respectively.

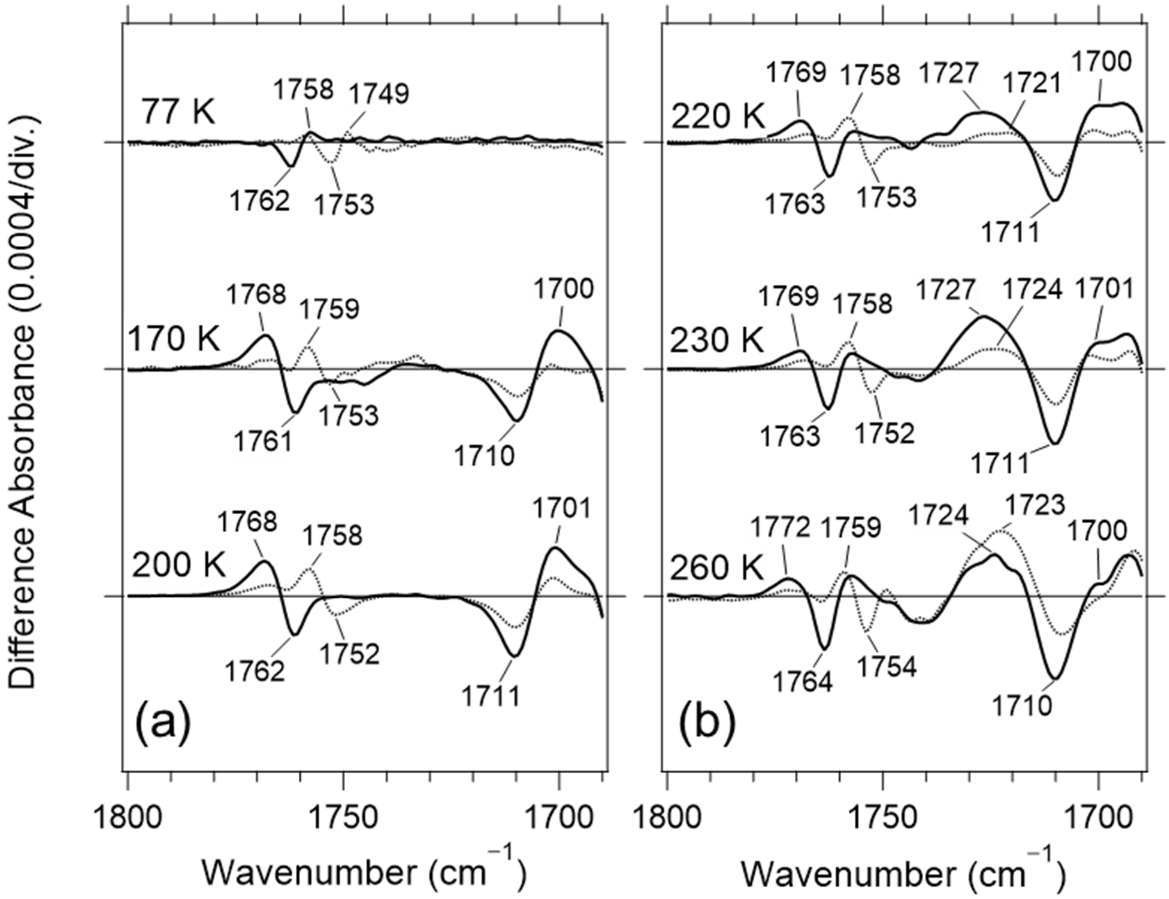

Figure S3. Light-induced FTIR difference spectra in the 1800–1690 cm^{-1} region recorded at 77, 170, and 200 K (a) and at 220, 230, and 260 K (b). Solid and dotted lines denote spectra measured under H_2O and D_2O hydration, respectively. One division on the y-axis corresponds to 0.0004 absorbance units in each panel.

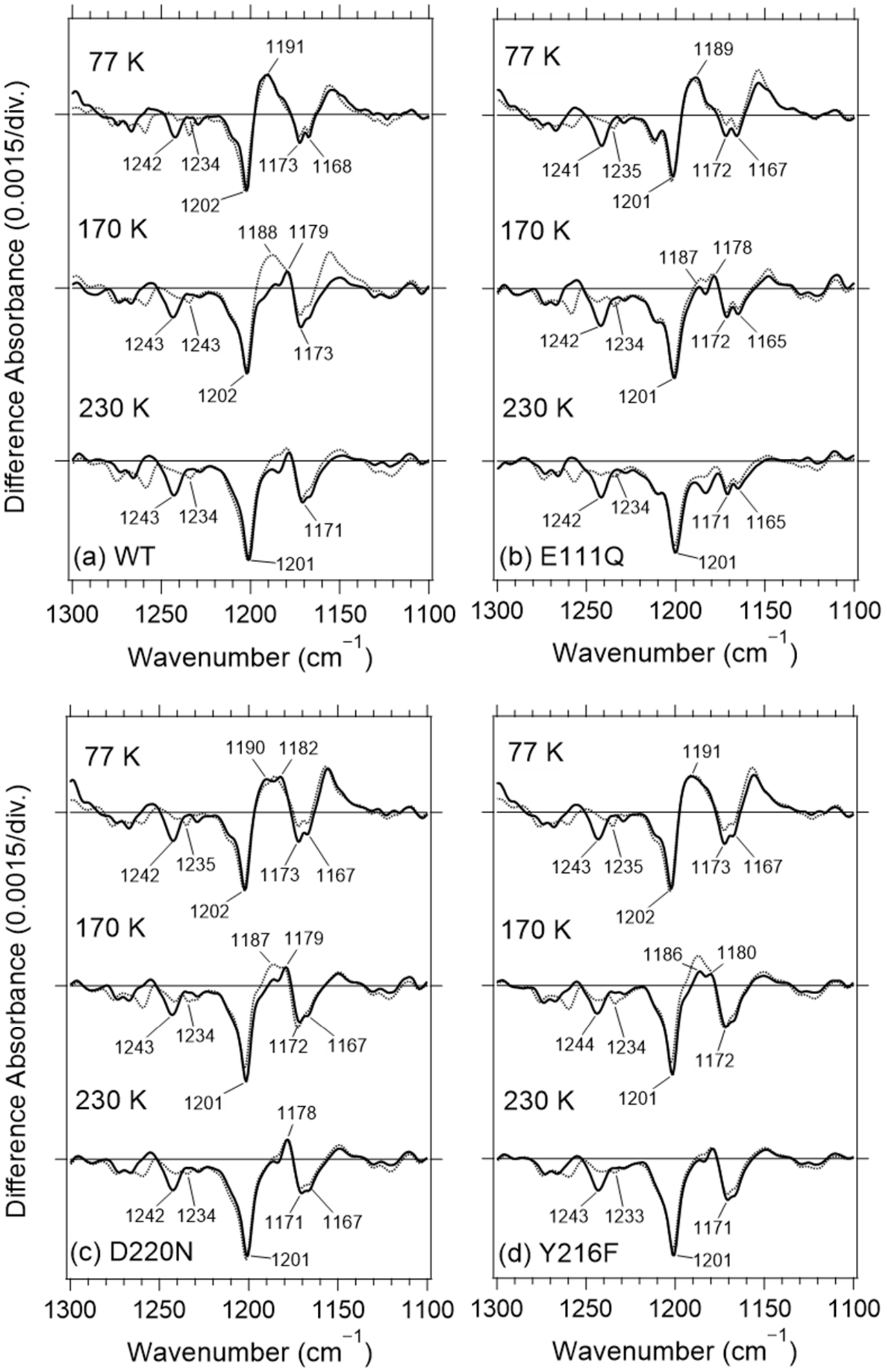

Figure S4. Light-induced FTIR difference spectra in the 1300–1100 cm^{-1} region recorded at 77, 170, and 230 K for WT (a), E111Q (b), D220N (c), and Y216F (d). Solid and dotted lines denote spectra measured under H_2O and D_2O hydration, respectively. One division on the y-axis corresponds to 0.0015 absorbance units in each panel.

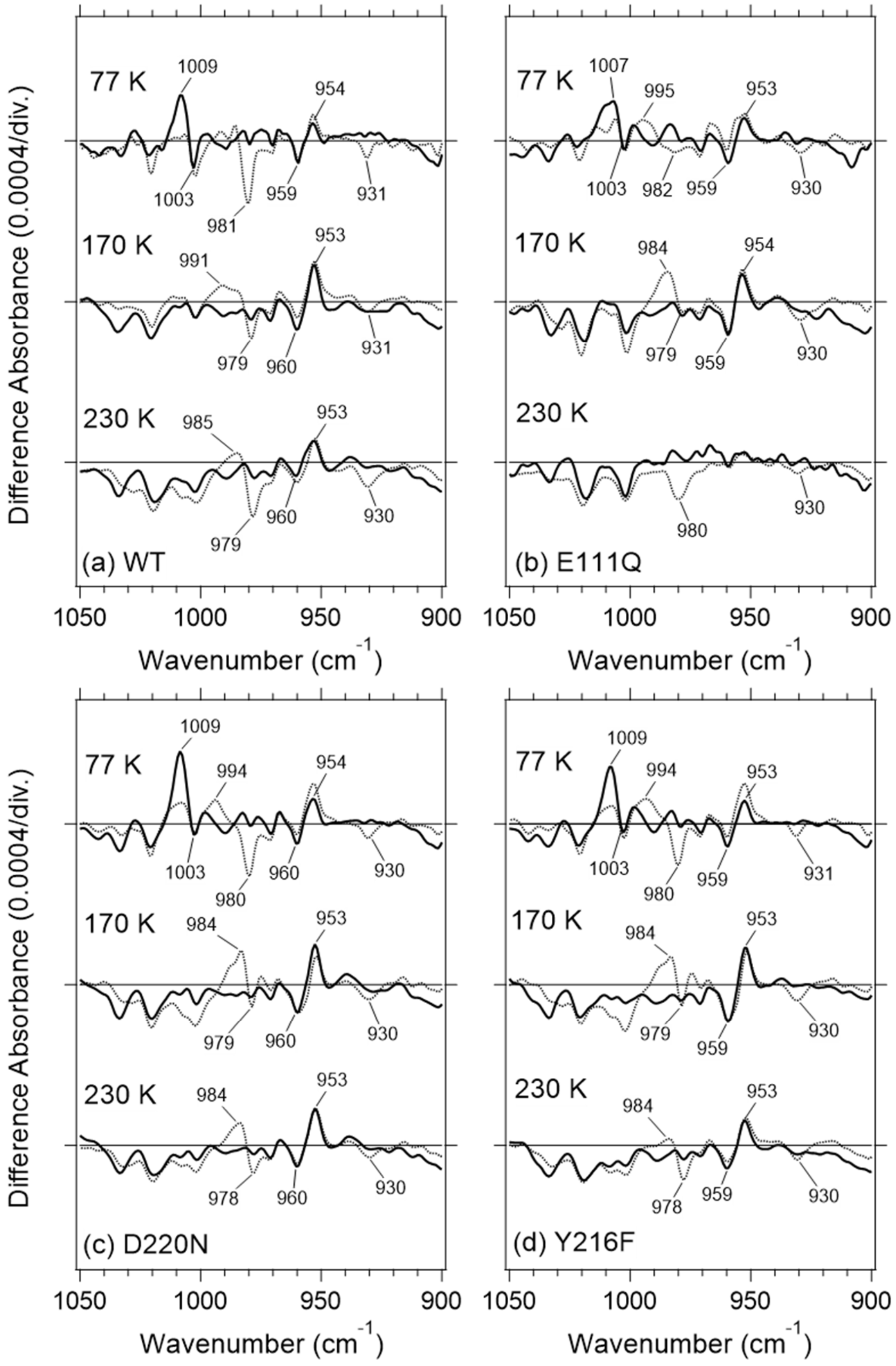

Figure S5. Light-induced FTIR difference spectra in the 1050–900 cm^{-1} region recorded at 77, 170, and 230 K for WT (a), E111Q (b), D220N (c), and Y216F (d). Solid and dotted lines denote spectra measured under H_2O and D_2O hydration, respectively. One division on the y-axis corresponds to 0.0004 absorbance units in each panel.

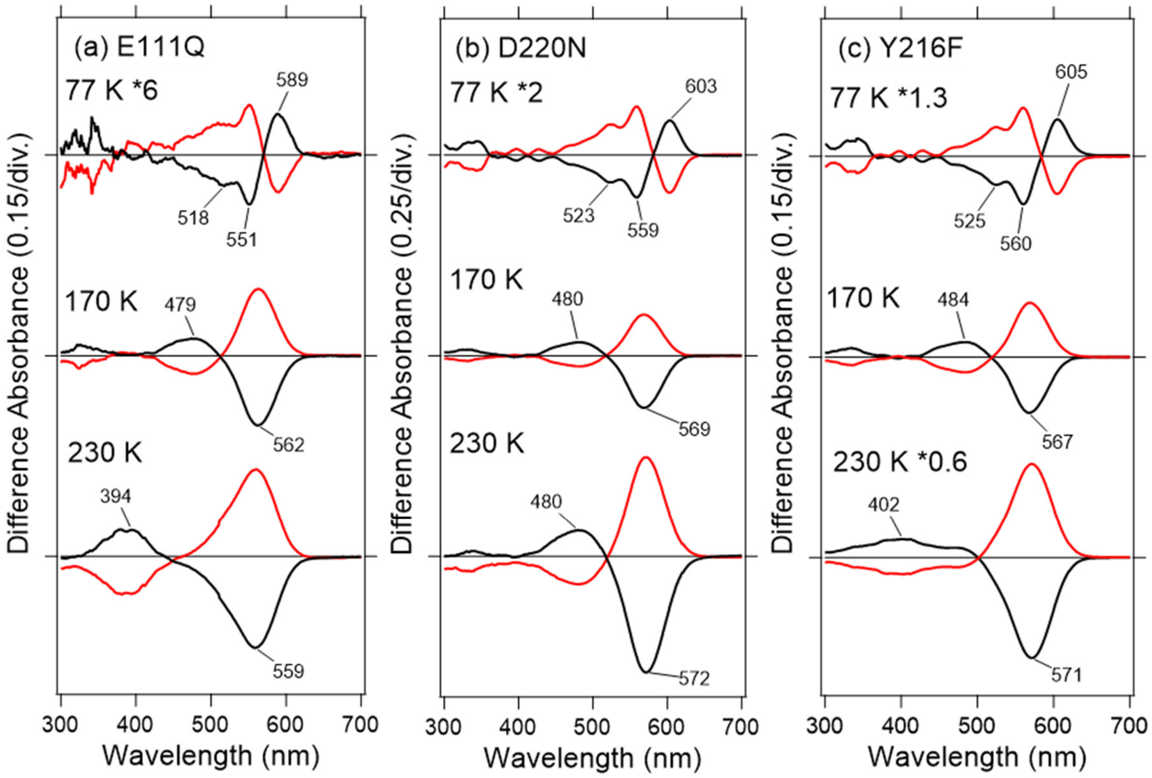

Figure S6. Light-induced difference visible absorption spectra of E111Q (a), D220N (b), and Y216F (c) mutants of *Ns*XeR recorded at 77, 170, and 230 K. The forward and backward photoreactions are represented by solid black and red lines, respectively. In each panel, the difference spectrum (black line) shows the negative and positive sides corresponding to the dark state and the red-shifted (K) or blue-shifted (L or M) intermediate, respectively. The difference spectrum (red line) in each panel represents the reverse reaction. The spectra of E111Q, D220N, and Y216F recorded at 77 K were normalized by factors of 6, 2, and 1.3, respectively. The spectra of Y216F recorded at 230 K were normalized by factors of 0.6.

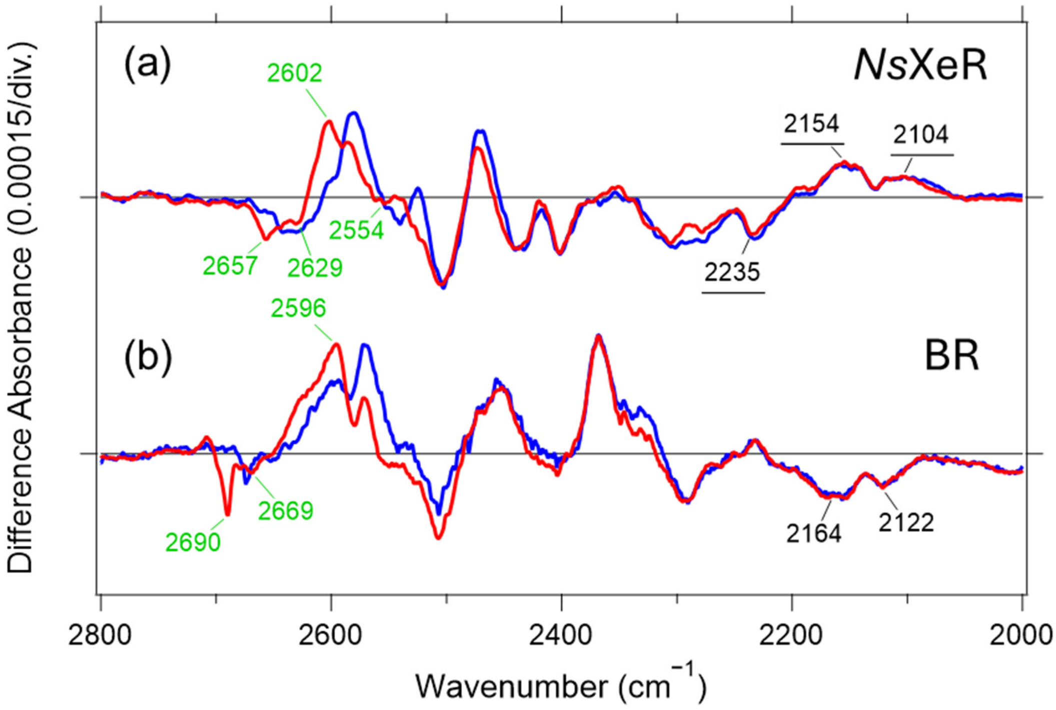

Figure S7. Comparison of light-induced difference FTIR spectra of BR and *Ns*XeR recorded at 170 K. The difference spectra of *Ns*XeR and BR are reproduced from Figure 4 in the main text and Figure 3 in the reference 18, respectively. Red and blue solid lines denote spectra measured under D_2O and $D_2{}^{18}O$ hydration, respectively. One division on the y-axis corresponds to 0.00015 absorbance units.

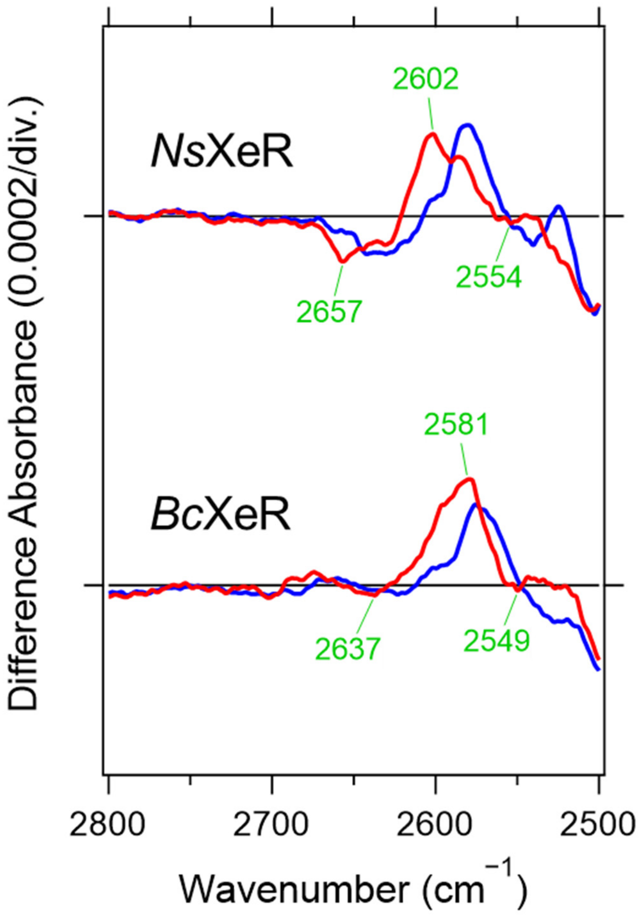

Figure S8. Light-induced FTIR difference spectra in the 2800–2500 cm^{-1} region recorded at 170 K for *Ns*XeR and *Bc*XeR. Red and blue solid lines denote spectra measured under D_2O and $D_2{}^{18}O$ hydration, respectively. One division on the y-axis corresponds to 0.0002 absorbance units.

Materials and methods used in this study

Preparation of the NsXeR and BcXeR samples.

The *Ns*XeR samples were prepared similarly to previous reports.[1-3] The *Bc*XeR sample was produced as described before.[4] For the expression of uniformly ^{15}N-labeled or ^{15}N-ε-lysine–labeled *Ns*XeR protein, the cultivation medium was replaced with M9 minimal medium (50 mM Na_2HPO_4, 25 mM KH_2PO_4, 10 mM NaCl, 1 mM $MgSO_4$, 10 mM Glucose, 3.3 μM $FeCl_3$, 50 μM $MnCl_2$, 80 μM Biotin, 60 μM Thiamine, 10 mM NH_4Cl). For the production of uniformly ^{15}N-labeled *Ns*XeR, $^{15}NH_4Cl$ was supplied as the sole nitrogen source. For the production of ^{15}N-ε-lysine–labeled *Ns*XeR, 100 mg/mL of ^{15}N-ε-lysine was added at the same time as isopropyl β-D-thiogalactopyranoside (IPTG) induction. Purified *Ns*XeR proteins were reconstituted into a lipid membrane composed of 1-palmitoyl-2-oleoyl-phosphatidylethanolamine and 1-palmitoyl-2-oleoyl-phosphatidylglycerol (molar ratio 3:1) at a protein-to-lipid molar ratio of 1:20. The reconstituted samples were resuspended in a buffer (1 mM NaCl and 2 mM Tris–HCl (pH 8.0)) and adjusted to a concentration of 2.5 mg mL^{-1}. A 60 μL aliquot was placed on a BaF_2 window and dried using an aspirator.

Low-temperature FTIR spectroscopy

For low-temperature FTIR measurements, *Ns*XeR films were performed according to the previous study.[2] The film samples were hydrated with H_2O, D_2O, or $D_2{}^{18}O$ before analysis. The hydrated sample was mounted on a sample holder and placed in a cryostat (Optistat DN2, Oxford Instruments) attached to an FTIR spectrometer (Cary 670, Agilent Technologies Japan, Ltd.). The sample was cooled to 77–260 K using liquid nitrogen, with temperature regulation at ±0.1 K precision. To generate the L and M intermediates, *Ns*XeR wild type (WT) was illuminated with >500 nm light (Y-52 cut-off filter, Toshiba) from a 300 W Xenon lamp (Max-303, Asahi Spectra) for 2 min. The L intermediate was reverted to the dark state by exposure to 470 nm light (Toshiba interference filter) for 2 min. The M intermediate was reverted to the dark state by exposure to 400 nm light (Toshiba interference filter for 2 min. For FTIR spectroscopy, interferograms were collected before and after photoreactions, each constructed

from 128 scans. Multiple irradiation sequences were averaged: 40 spectra for H_2O-hydrated *Ns*XeR and 100 spectra for D_2O- and $D_2{}^{18}O$-hydrated samples.

References

(1) Shevchenko, V.; Mager, T.; Kovalev, K.; Polovinkin, V.; Alekseev, A.; Juettner, J.; Chizhov, I.; Bamann, C.; Vavourakis, C.; Ghai, R.; et al. Inward H(+) pump xenorhodopsin: Mechanism and alternative optogenetic approach. *Sci. Adv.* **2017**, *3* (9), e1603187.
(2) Ito, Y.; Nishikino, T.; Kandori, H.; Furutani, Y. The role of retinal chromophore photoisomerization in enhanced inward proton-pumping activity of xenorhodopsin from *Nanosalina*. *Biochim. Biophys. Acta Bioenerg.* **2025**, *1866* (3), 149556.
(3) Vlasova, A.; Polyakova, A.; Gromova, A.; Dolotova, S.; Bukhalovich, S.; Bagaeva, D.; Bondarev, N.; Tsybrov, F.; Kovalev, K.; Mikhailov, A.; et al. Optogenetic cytosol acidification of mammalian cells using an inward proton-pumping rhodopsin. *Int. J. Biol. Macromol.* **2023**, *242* (Pt 3), 124949.
(4) Kovalev, K.; Tsybrov, F.; Alekseev, A.; Shevchenko, V.; Soloviov, D.; Siletsky, S.; Bourenkov, G.; Agthe, M.; Nikolova, M.; von Stetten, D.; et al. Mechanisms of inward transmembrane proton translocation. *Nat. Struct. Mol. Biol.* **2023**, *30* (7), 970-979.
